# Physicochemical and Rheological Characterization of Different Low Molecular Weight Gellan Gum Products and Derived Ionotropic Crosslinked Hydrogels

**DOI:** 10.3390/gels7020062

**Published:** 2021-05-26

**Authors:** Calogero Fiorica, Giuseppina Biscari, Fabio Salvatore Palumbo, Giovanna Pitarresi, Annalisa Martorana, Gaetano Giammona

**Affiliations:** Department of “Scienze e Tecnologie Biologiche, Chimiche e Farmaceutiche” (STEBICEF), University of Palermo, Via Archirafi, 32, 90123 Palermo, Italy; giuseppina.biscari@unipa.it (G.B.); fabiosalvatore.palumbo@unipa.it (F.S.P.); giovanna.pitarresi@unipa.it (G.P.); annalisa.martorana@unipa.it (A.M.); gaetano.giammona@unipa.it (G.G.)

**Keywords:** low molecular weight gellan gum, basic hydrolysis, ionotropic crosslinked gellan gum hydrogels, thermotropic behavior

## Abstract

A series of four different low molecular weight gellan gum products was obtained by alkaline hydrolysis with the aim to investigate the impact of the molecular weight on the rheological properties of the polysaccharide aqueous dispersions and on the physicochemical characteristics of derived ionotropic crosslinked hydrogels. In particular, thermo-rheological analysis was conducted on aqueous dispersions to study the influence of molecular weight on the thermogelation properties typical of the native polysaccharide while strain sweep experiments were conducted to establish if aqueous dispersion shows a viscoelastic behavior. The effect of different Ca^2+^ on the rheological properties of hydrogels were studied. Furthermore, ionotropic crosslinked hydrogels were analyzed in terms of morphology on the dried state and swelling behavior, while their viscoelastic properties were studied by means of rheological analysis conducted in frequency sweep regime after different time points of incubation in phosphate buffer at pH 7.4. Release experiments conducted using fluorescein isothiocyanate labelled dextran as a model diffusion agent and was performed to investigate the possibility of using the low molecular weight GG-derived hydrogels as an active molecule-releasing device. Finally, the cytocompatibility of hydrolysis products was investigated, as well as the capacity of hydrogels to encapsulate viable MC3T3-E1 preosteoblastic cells.

## 1. Introduction

Gellan Gum (GG) is a high molecular weight bacterial exopolysaccharide secreted by Pseudomonas elodea during aerobic fermentation. It is an anionic polysaccharide composed of a tetrasaccharide repeating unit of one α-L-rhamnose (Rhap), one β-D-glucuronic acid (GlcpA) and two β-D-glucoses (Glcp) [1]. As a food additive, GG has been used as a thickener, gelling agent and stabilizer in many products of the food industry. Because of its low cost and biological properties, such as low cytotoxicity and biodegradability, and because of its peculiar physicochemical characteristics, GG has been largely studied as a material for biomedical applications [2,3].

Two types of GG have been mainly used for these purposes. The former is characterized by the presence of acyl substituents and is known as high-acyl, the second is called low acyl or deacetylated and is obtained by alkaline hydrolysis of HA Gellan Gum [4].

Both forms are soluble in hot water and, as the temperature decreases, tangled polymer chains undergo a coil-to-double helix transition that gives rise to the obtainment of both soft or brittle hydrogel, depending on the acyl content of the starting polysaccharide [5]. This temperature-induced gelation is due to the establishment of weak interactions, such as hydrogen bonds and van der Waals forces between the polymeric chains that promote the obtainment of double helical conformation.

Besides the temperature-induced gelation, GG can be further crosslinked in the presence of both mono or bivalent cations. Cations, such as Ca^2+^ or Mg^++^, compared to monovalent ions, induce a more effective crosslinking process since they shield the electrostatic repulsion between the double helices and form coordination physical bonds with GG carboxylate groups inducing an intimate aggregation of the ordered anionic double-helices that will in turn form a three-dimensional hydrated network [6].

Despite the numerous appealing properties, GG shows some serious drawbacks that limit its applicability for several biological uses. For example, because of the high molecular weight, GG need a stressed heating process to be dispersed and water (90 °C for 30 min). Moreover, even at relatively low concentrations (0.5–1% *w/v*), it forms too viscous aqueous dispersions, which quickly undergo gelation and lost fluidity as a consequence of temperature lowering. For these reasons, “native” polysaccharide cannot be used, for example, for cell encapsulation, nor for thermolabile products [7].

Two main strategies can be adopted to overcome these limitations. One relies on the chemical modification of the polysaccharide backbone, while the other one concerns the reduction of GG molecular weight. Often, these two strategies are used in combination to obtain injectable GG-based aqueous dispersions that form a hydrogel in contact with body fluids. Pendant moieties, inserted to the polysaccharide backbone exploiting both hydroxyl and carboxyl groups, can perturbate the coil-to-helix transition facilitating the obtainment of gelling precursor that are fluid at room temperature but still sensitive to the external medium ionic strength [8,9,10,11,12].

Concerning the reduction of GG molecular weight, hydrolysis (both acid or alkaline) conducted in aqueous condition allows reproducible results [13,14].

Although there are several studies describing the characterization of low molecular weight GG products, mainly by investigating the rheological properties of aqueous dispersion [15,16,17], a study on the correlation of the rheological properties of different low molecular weight GG with the physicochemical properties of derived ionotropic crosslinked hydrogels has not been carried out so far.

Low molecular weight GG products were obtained by alkaline hydrolysis of a high molecular weight LAGG varying the reaction times, while keeping the temperature constant.

The aim of this work was to investigate whether the drastic reduction of GG molecular weight still allows the obtainment of thermotropic products that can be crosslinked, exploiting the ionic strength of the external medium. Furthermore, we aimed to investigate the influence of the molecular weight on the main physicochemical features of GG hydrogels.

## 2. Results and Discussion

Basic hydrolysis is a well-known method used to reduce GG molecular weight in order to obtain derivatives that can be dispersed in water at relatively high concentrations without resulting in too viscous solutions, still maintaining ionotropic and thermos-rheological properties such as those of native GG.

**Figure 1:**
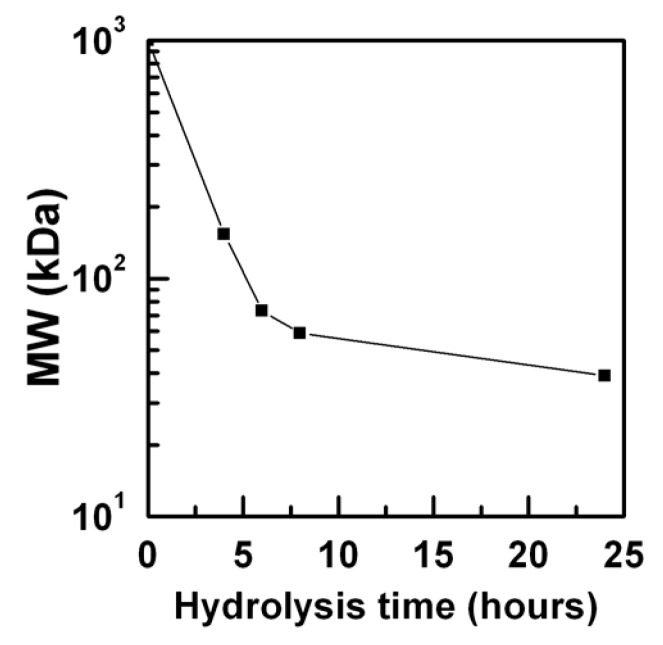
Reduction of GG molecular weight as a function of basic hydrolysis time.

Here, with the aim to obtain low molecular weight products, GG was hydrolyzed in drastic conditions, dispersing it at a concentration of 1% *w/v* in NaOH 0.1 N solution, maintaining a constant temperature of 50 °C. By changing the reaction time, we obtained four products with different low molecular weight and PI, listed in Figure 1 and Table 1.

From data reported, it is possible to notice that PI decreases proportionally with the MW, meaning that the hydrolysis process uniforms the size of degraded macromolecular chains. Considering that the starting GG has an MW of 1000 kDa (product supplier information), it is clear that the hydrolysis occurs mainly during the first 6 h, since the MW is reduced by an order of magnitude, and became slower after this time until 24 h. The product named GG_24_ resulted in being water-dispersible, even at room temperature, and until a concentration of 5% *w/v*, while for a higher concentration it needs higher temperatures (≥50 °C). For all the other products, it was possible to produce aqueous dispersions with concentrations ranging from 5% to 10% *w/v* by means of oven incubation at 80 °C.

Interestingly, all the hydrolysis products resulted in being freely dispersible in dimethyl sulfoxide at 50 °C and at a concentration of 1% *w/v*, producing a clear dispersion already after 1 h of magnetic stirring. This aspect is interesting since the poor dispersibility of a high molecular weight GG in this organic solvent often brings the necessity of producing its tetrabutylammonium salt to perform chemical functionalizations that cannot be performed in an aqueous environment [8,18]. Although this procedure is simple to conduct, it still requires several time-consuming steps that could be bypassed using GG with lower molecular weights.

Among the physicochemical properties of GG aqueous dispersions, thermotropic behavior is particularly interesting, since it allows a thermally reversible coil-to-helix transition triggered by the temperature decrease that leads to the formation of physical hydrogels at room temperature. Several studies demonstrate that this transition is promoted with increasing molar mass, concentration of the starting dispersion and cation presence [15,16,19].

High molecular weight GG aqueous dispersions form stable physical hydrogels at temperatures below 40 °C, even at a low concentration.

**Figure 2:**
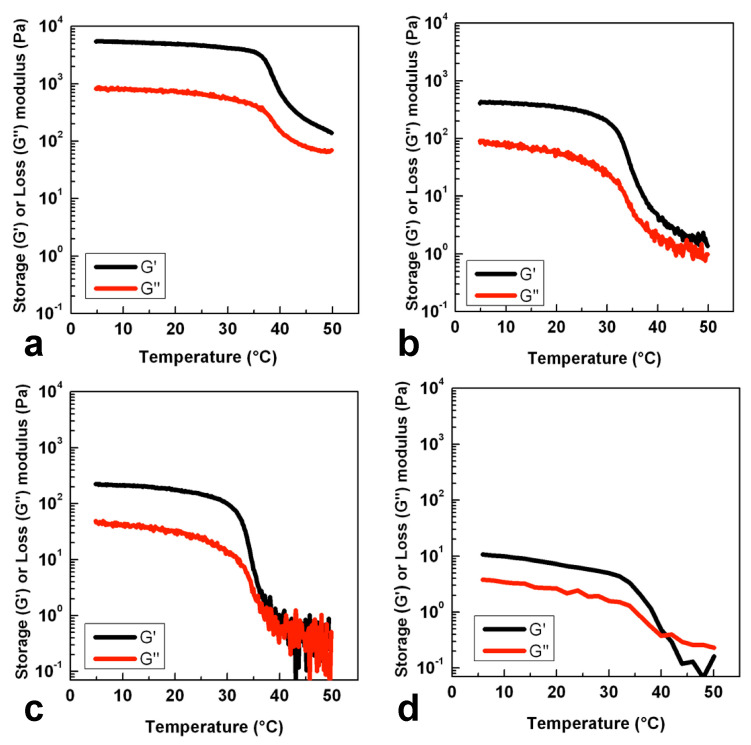
Temperature dependence of storage modulus (G) and loss modulus (G′′) during cooling process for hydrolysis product GG_4_ (**a**), GG_6_ (**b**), GG_8_ (**c**) and GG_24_ (**d**) dispersed in water at 5% *w/v*. Experiments were performed at 0.5 Hz and 1% of strain.

It is possible to notice from Figure 2 that aqueous dispersions at 5% *w/v* for all the obtained low molecular products show the typical thermotropic behavior of GG, since during the cooling process a sensible increase in both storage (G′) and loss (G″) modulus can be observed. For all the investigated samples, the temperature at which G′ reached the plateau was below 40 °C with no significative changes between different samples. G′ values at a low temperature decreased proportionally with the sample molecular weight (with no significative differences between GG_6_ and GG_8_), demonstrating that, at this concentration, the coil-to-helix aggregation for longer macromolecular chains leads to the formation of a major number of junction zones, which in turn determine the obtainment of stiffer physical hydrogels.

At room temperature, GG_24_ aqueous dispersion (5% *w/v*) forms a loose hydrogel that does not flow under the influence of gravity, following the inversion of the test tube, but begins to flow when slightly disturbed. Interestingly, this dispersion maintains its ionotropic properties since it forms a stable hydrogel when in contact with DPBS pH 7.4 or with CaCl_2_ 0.1 M aqueous solution. Clearly, cations induce the establishment of coordination bonds that strengthen the hydrated three dimensional network. Overall, this behavior is particularly interesting in the perspective of developing injectable biomedical systems whose gelation is induced by contact with physiologic fluids.

**Figure 3:**
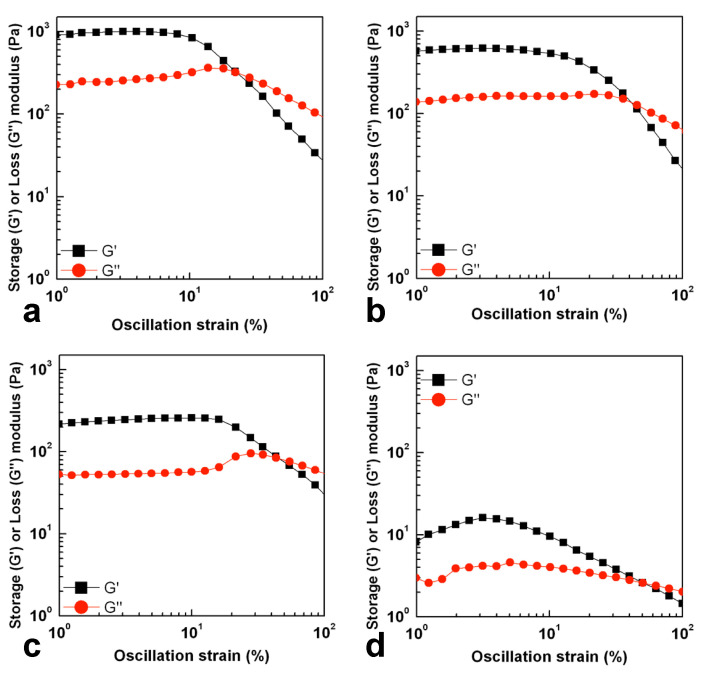
Strain sweep experiments performed at 0.1 Hz for hydrolysis product GG_4_ (**a**), GG_6_ (**b**), GG_8_ (**c**) and GG_24_ (**d**) dispersed in water at 5% *w/v*.

Molecular weight and molecular weight distribution influence the rheological behavior of polymer solutions. Normally in rheological analysis, small amplitude oscillatory shear (SAOS) is used to research the linear viscoelastic region, which however fails in characterizing the material structure, processing, applications, and functions. The rheological properties of hydrolyzed GG samples here produced were investigated using a large amplitude oscillatory shear (LAOS) technique to provide an explanation of the microstructural differences between different molecular weight GG. To describe these proprieties, an LAOS technique was found to be very sensitive to explore the polymer interactions in a non-linear viscoelastic regime. In the linear viscoelastic regime (LVE), the storage and loss moduli, G′ and G″, respectively, are nearly parallel in indicating LVE behavior with a decrease in both moduli as the molecular weight decreases. As the strain increases, reaching the critical strain, samples undergo a transition where G’ decreases suddenly as the strain increases, while G″ first increases and then decreases. This behavior is defined as a weak strain overshoot (LAOS type III) [20] and it is shown in the GG_4_, GG_6_ and GG_8_ samples. As illustrated in Figure 3 the samples with a higher molecular weight require a smaller critical strain to disrupt the equilibrium microstructure than those with a lower molecular weight. This can be due to the balance between the formation and the destruction of the network junctions [21]. The formation of weak interactions stabilize the double helix and this causes a more evident strain overshoot behavior. Tong et al. [22] used a LAOS technique to show the microstructural differences between HA and LAGG. They showed a more pronounced strain overshoot behavior for HA gellan gum with respect to LAGG due to the stabilization of the double helix structure due to the presence of glycerate group in HA gellan gum.

In high molecular weight samples, the chains, in aqueous solutions at 25 °C, assume a helix conformation and the chains are highly extended due to the electrostatic repulsion from the charged groups on the side chains. When a deformation is applied, the molecules may align and, up to a certain strain, G″ slightly increases. When a large deformation is applied, over the critical strain, the complex structure is destroyed, after which the polymer chains align with the flow field, and G″ decreases. The relative intensity of the overshoot and the strain value at maximum G’’ decreases with the increase in molecular weight. Indeed, this behavior is more evident for GG_8_ while it is less pronounced for GG_4_ and GG_6_ (Figure 3), showing the strong impact of the molecular weight on the sample microstructure interactions. This can be probably explained assuming that as the molecular weight increases, chains are forced together to be closest and the electrostatic repulsion can be stronger than the capacity to form weak interactions, resulting in a faster disruption of the microstructure for GG_4_ and GG_6_ compared to GG_8_.

The molecular weight highly influences the coil-to-helix transition during gelation. In particular, the transition is promoted with increases in the molar mass. Considering the GG_24_ sample, double helix formation occurred as shown in thermo-rheological analysis (Figure 2d) and as already studied by Ogawa et al. [23] They evaluated the effect of molar mass on the coil-to-helix transition, concluding that the lowest molar mass below which no helix is formed at 25 °C in aqueous solutions with 25 mmol NaCl, lies between *M*w = 32 × 10^3^ and *M*w = 17 × 10^3^, i.e., below the molecular weight of GG_24_. Although the double-helix is obtained, the molecular weight may be too low to stabilize the double helix. This may cause a continuous rearrangement of the microstructures during oscillatory shear, leading to a slight increase of both moduli at a low strain % for GG_24_. When the critical strain is reached, the chains align with the flow and both moduli decrease. To summarize, the molecular weight largely impacts the balance between microstructure interactions.

Ionotropic crosslinking was studied by inducing the sample gelation in the presence of two different amounts of CaCl_2_. For these analyses, samples were dispersed in MilliQ water and the salt solution was added to the hot polymeric dispersion. The GG concentration in this case was set to 2% *w/v* (lower compared to the just described rheological analyses) to avoid quick and inhomogeneous gelation that can occur in the concentrated hot dispersion following the addition of the salt solution.

**Figure 4:**
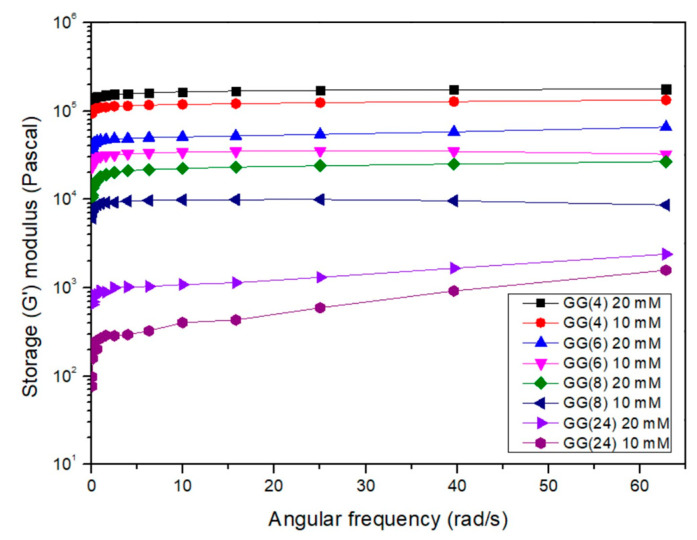
Elastic moduli (G’) obtained from frequency sweep rheograms performed at 1% of strain on GG ionotropic crosslinked hydrogels with different CaCl_2_ concentrations.

It is possible to notice from Figure 4, that G’ values decrease as the molecular weight of GG decreases and show an increment in accordance with the CaCl_2_ concentration, confirming that all the obtained hydrolysis products retain the typical ionic strength sensitivity of a high molecular weight GG.

For morphological, physicochemical and rheological studies, crosslinked samples were produced by curing the temperature-induced hydrogels (obtained from 5% *w/v* aqueous dispersion) with a CaCl_2_ 0.1 M solution. The obtained samples were fractured on the swollen state and freeze-dried to investigate the morphology of xerogels by SEM studies.

**Figure 5:**
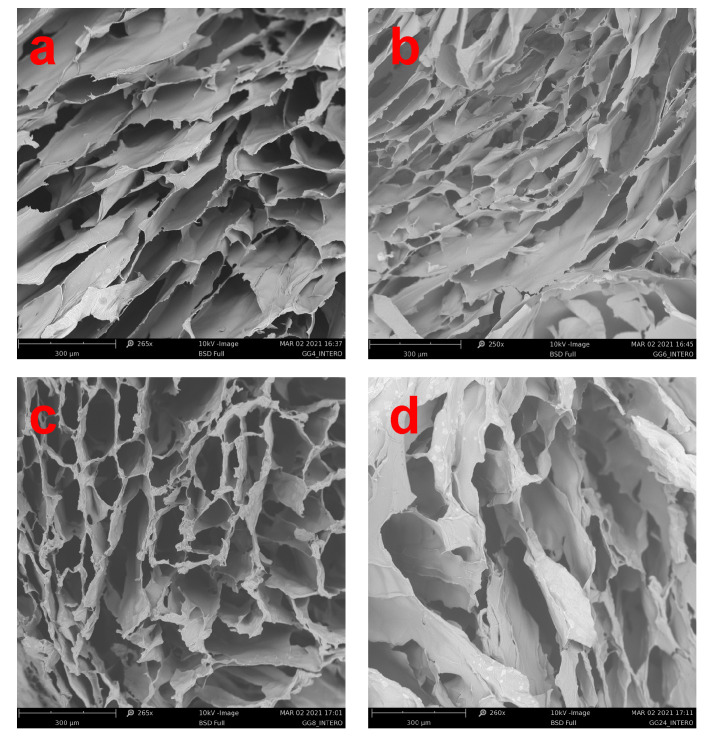
SEM images of freeze-dried hydrogel obtained by the ionotropic crosslinking of 5% *w/v* aqueous dispersion of GG_4_ (**a**), GG_6_ (**b**), GG_8_ (**c**) and GG_24_ (**d**).

Figure 5 reveals how it was predictable that all the freeze-dried products show a highly porous structure even if a less compact structure with larger pores is observable for GG_24_.

**Figure 6:**
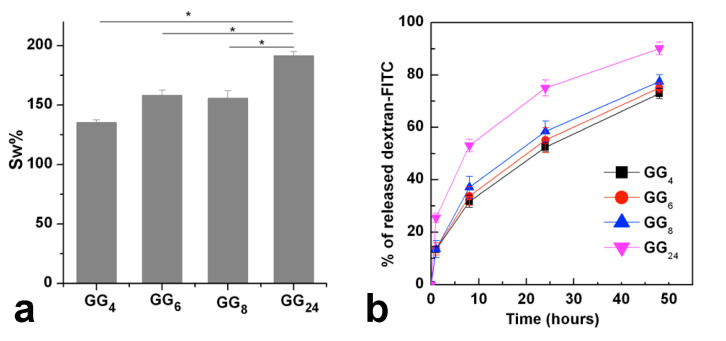
Swelling % of ionotropic crosslinked low molecular weight GG hydrogels incubated for 24 h in DPBS pH 7.4 at 37 °C (**a**), release of dextran- FITC from hydrogels (**b**).

These data are in accordance with those obtained from swelling studies that, besides revealing that all the investigated sample show a high capacity of incorporate aqueous medium, also demonstrate that GG_24_ hydrogel, due to its less-dense network, swells significantly more compared with the hydrogels produced, starting from products with a higher molecular weight (Figure 6a).

The release of dextran-FITC loaded into the ionotropic crosslinked hydrogels demonstrate that the diffusion of the labelled polysaccharide is quicker from the hydrogel obtained from GG_24_, likely because of the higher hydration of the polymeric network. No significative differences in the diffusion rate where observable form hydrogels obtained from GG_4_, GG_6_ and GG_8_ (Figure 6b).

All the samples reach the swelling equilibrium at 37 °C after 24 h of incubation (no significative differences were observed in the sample weight after 48 h).

At scheduled incubation times, the viscoelastic properties of ionotropic crosslinked hydrogels were also investigated by means of rheology tests performed in a frequency sweep.

The curing with CaCl_2_ 0.1 M for 1 h allows the formation of stable physical hydrogels, which can be easily handled. These samples were incubated in DPBS pH 7.4 to mimic the physiologic conditions where the exchange of calcium ions with a monovalent one causes the loosening of the hydrogel structures.

Rheological analyses were conducted at scheduled time points to measure the effect of calcium depletion onto the elastic properties of samples with the aim to investigate if this effect can be influenced by the polysaccharide starting molecular weight.

**Figure 7:**
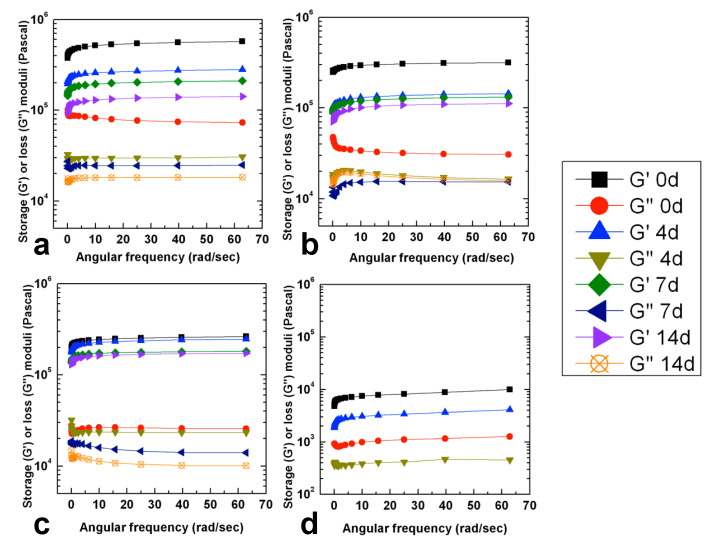
Frequency sweep rheograms performed at 1% of strain for GG_4_ (**a**), GG_6_ (**b**), GG_8_ (**c**) and GG_24_ (**d**) ionotropic crosslinked hydrogels incubated for different times in DPBS pH 7.4 at 37 °C.

**Figure 8:**
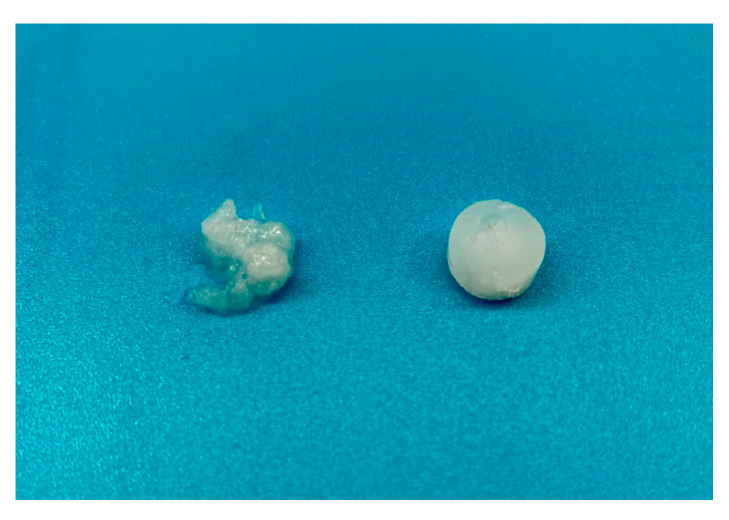
GG24 (on the right) and GG8 (on the left) ionotropic crosslinked hydrogels after 1 week of incubation at 37 °C in DPBS pH 7.4.

As shown in Figure 7, the value of samples G′ modulus, measured soon after the ionotropic crosslinking procedure, decreases as the molecular weight of sample decreases in accordance with what was already observed in previous analyses. A decrement in the G′ values was observed for all the investigated samples as the incubation time increases, demonstrating that Ca^2+^ loss actually causes the loosening of the polymeric network. It is possible to notice that, except for GG24, after 14 days of incubation, the G’ value is similar for GG4, GG6 and GG8 and shows values around 10^5^ Pascal. These results can reflect the hydrogels stability. Macroscopically, except for hydrogels obtained starting from the product with the lower molecular weight (GG_24_), samples retained their structural integrity during all the investigated time points, while hydrogels obtained with the GG_24_ product started to appear fragmented already after one week of incubation and it was not possible to handle them to perform a repeatable rheological analysis (Figure 8).

Cytocompatibility tests were conducted using MC3T3-E1 pre osteoblastic cells, chosen as a model cell line.

**Figure 9:**
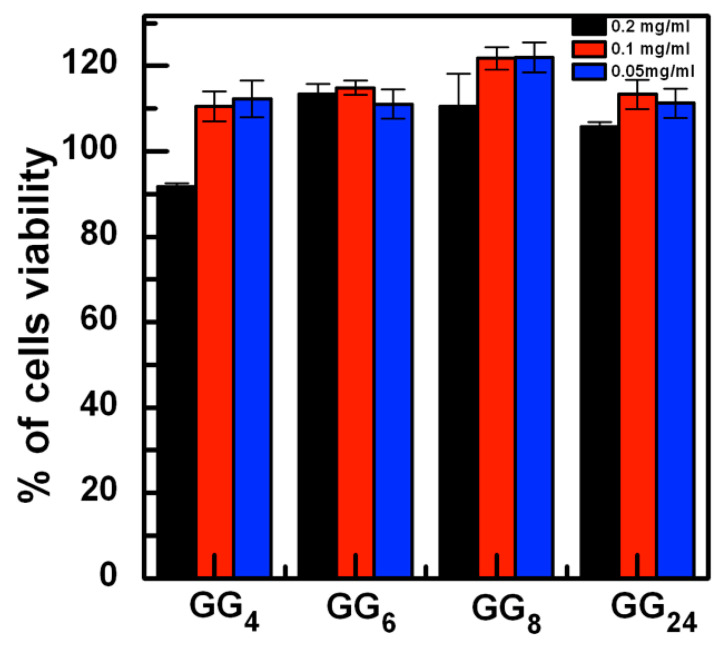
Viability of MC3T3-E1 preosteoblastic cells cultured for 24 h in the presence of GG degradation products at different concentrations. Viability is expressed as % compared to untreated cells.

It is possible to notice that, for almost all the investigated samples and at all the concentrations tested, a slight increase in cell viability compared to the control was observed after 24 h of incubation. No significant differences were noticed between different samples or between different concentrations, except for GG4 at 0.2 mg/mL, which shows a significant (*p* < 0.05) decrease in cell viability compared to its lower concentrations or to other investigated samples (Figure 9). It is likely that, for this sample, the formation of coarse particles that settle onto the cells can cause a slight decrement in the cell growth. In any case, the viability for this sample is higher than 90%.

By dispersing GG hydrolysis products at a concentration of 2.5 *w/v*, it was possible to obtain at room temperature fluid systems that can be easily loaded onto 1 mL syringe and mixed with the cell suspension to produce cell-encapsulated hydrogels. Upon mixing, the culture medium induces an increase in the dispersion viscosity into the syringe due to a partial gelation of the systems. In any case, it was still possible to easily extrude the cell containing hydrogel into the culture wells. Encapsulated cells were cultured for 24 h and then treated with a live and dead fluorescence staining kit that allows us to observe both viable (green) and dead (red) cells.

**Figure 10:**
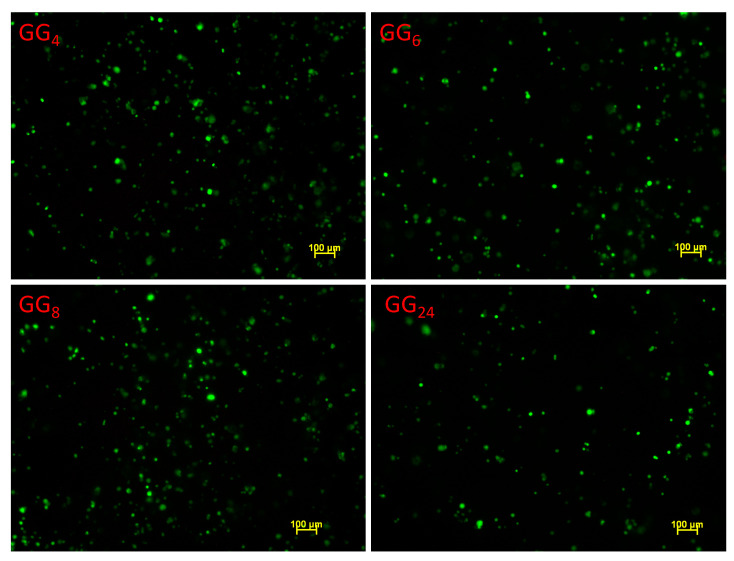
Live/dead staining of MC3T3-E1 preosteoblastic cells encapsulated into GG products after 24 h of culture.

In all the investigated hydrogels, no dead cells were observable, confirming that neither the encapsulation process nor the material itself had a negative effect on the cell viability (Figure 10).

## 3. Conclusions

To make the most of the potential physicochemical properties of GG and to make it easily processable through all the main biofabrication techniques, various strategies have been used so far, such as its chemical functionalization or mixture with other biomaterials. To overcome some of the inadequate properties of the high molecular weight polysaccharide, in this work, we focused on the production of different low molecular weight GG derivatives, which can be more manageable and versatile from a technological point of view. As stated above, the reduction of GG molecular weight is advantageous to obtain products that result in being more easily processable since they can be dispersed in water at relatively high concentrations without being too stressed in heating conditions and avoiding the formation of too-viscous solutions. Alkaline hydrolysis of GG was conducted at 50 °C in NaOH 0.1 N. By varying the degradation time, four different GGs with molecular weights ranging from 149 to 36 kDa were obtained. By means of thermorheological analysis, conducted on 5% *w/v* aqueous dispersions, it was demonstrated that all the obtained low molecular weight products maintained the typical temperature responsiveness of high molecular weight GG since they underwent a cooling-induced gelation mediated by the coil-to-helix transition of the polysaccharide chains. The obtained products resulted in being easily dispersible in DMSO, facilitating those synthetic routes that make it possible to functionalize GG hydroxyl groups in spite of the carboxylic ones. Considering that the carboxylate groups play a crucial role in the gelation mechanism, this aspect could be extremely interesting. Ionotropic gelation of the aqueous dispersions, conducted by treatment with CaCl_2_ with two different procedures, demonstrates that low molecular weight products retain the ability to crosslink as a function of the medium ionic strength. The crosslinked procedure leads to the formation of mechanically stable hydrogels characterized by a highly porous structure on the micrometric scale, as demonstrated by SEM images of the freeze-dried samples. These low molecular weight GG hydrogels can uptake high amounts of aqueous medium already after 24 h of incubation. Except for the sample named GG_24_ having a molecular weight of 39 kDa, all the investigated samples retained their viscoelastic properties in a physiological environment at 37 °C for at least 14 days.

All the hydrolysis-derived GG samples resulted in being cytocompatible. The reduction in molecular weight allows obtained fluid dispersion that can be exploited to encapsulate viable cells and potentially release them in vivo.

On the whole, these studies demonstrated that despite the drastic reduction of the molar mass, the hydrolyzed GG maintains the peculiar rheological and physicochemical features of high molecular LAGG and can be an interesting and intelligent alternative to it in the development of biomedical devices alone or in combination with other biopolymers.

## 4. Materials and Methods

### 4.1. Chemicals and Apparatus

Gellan Gum (Gelrite^®®^), sodium hydroxide (NaOH), tetramethylammonium chloride (TMACl), live dead staining kit were purchased from Sigma-Aldrich, Milan, Italy.

Fluorescein labelled dextran Anionic, Lysine Fixable (Dextran-FITC) (Mw 10 kDa) was purchased by Thermo Fisher Scientific, Milan, Italy.

MTS reagent (CellTiter 96^®®^ AQueous One Solution Cell Proliferation Assay) was purchased from Promega, Milan, Italy.

Hydrolysis was conducted using rotating heating plate equipped with an independent temperature control/detection system.

Size exclusion chromatography was conducted with an Agilent 1260 Infinity multi-detector GPC/SEC system.

SEM analysis was conducted using a Phenom XL by Alfatest microscope operating at 5 kV (Alfatest, Rome, Italy).

The rheological tests were carried out using a DHR-2 oscillatory rheometer equipped with a self-heating Peltier plate (TA instrument, Sesto San Giovanni, Italy). 

Release studies were conducted with an UV-Vis spectrophotometer UV-240PC (Shimadzu, Salerno, Italy).

Cell cultures were performed using an Eppendorf New Brunswik S41i incubator, the viability was measured with Eppendorf AF2200 spectrophotometer.

Fluorescence images were obtained with AxioVert200 microscope (Zeiss, Milan, Italy).

### 4.2. Production of Low Molecular Weight GG

Basic hydrolysis of GG was performed as previously reported with slight modifications [10,24]. Briefly, GG was dispersed in 0.1 N NaOH solution at a concentration of 1% *w/v* and kept at 50 °C using a rotating heating plate. A blade stirrer (200 rpm for 10 min) was used to disperse coarse particles and allow the hydration of the powder. At scheduled time points (4, 6, 8 and 24 h), the pH of the dispersion was kept at 7 by adding HCl 1 N, the dispersion was cooled down at room temperature and dialyzed against MilliQ (spectrapore RC membrane with cut-off 50 kDa) for at least 5 days. Based on the hydrolysis time, the obtained samples were named GG_4_, GG_6,_ GG_8,_ and GG_24_.

### 4.3. Size Exclusion Chromatography Analysis (SEC)

The absolute weight-average molecular weight (Mw) and polydispersity index (PDI) of hydrolyzed GG products were measured by SEC using 0.025 M TMACl aqueous solution as a mobile phase and a Polysep P-4000 (Phenomenex) column as a stationary phase. The elution was conducted at 50 °C and with a flow rate of 0.8 mL/min using 20 kDa PEG as an internal standard. Before the elution, all the investigated samples were dissolved at 50 °C in the mobile phase.

### 4.4. Thermo-Rheological and Strain Sweep Experiments on Low Molecular Weight GG Aqueous Dispersions

To conduct a thermo-rheological analysis, samples were dispersed in MilliQ water at a concentration of 5% *w/v*. Clear homogeneous dispersions were obtained by placing the samples at 90 °C for 15 min. A parallel-plate geometry of 20 mm diameter was used for the experiments. Temperature dependence of the storage modulus (G′) and loss modulus (G″) values was analyzed by cooling the samples from 50 °C to 5 °C at a rate of 2 °C/min by applying a constant strain of 1% and a frequency of 0.5 Hz (3.14 rad/sec). Samples were previously equilibrated at a temperature of 50 °C for 60 s and a pre-shear of 0.01 Hz for 10 s was performed. The linear viscoelastic region was preliminarily assessed at both 50 °C and 5 °C by strain sweep experiments, applying a constant frequency of 0.5 Hz in the range between 0.5–40% of deformation.

To the already described aqueous dispersions, strain sweep experiments were conducted at a constant frequency of 0.1 Hz (0.68 rad/sec) in the range from 0.01 to 100 of strain %.

### 4.5. GG Hydrogels Production, Morphological Study and Evaluation of Viscoelastic Properties

Ionotropic crosslinking was carried by dispersing GG samples in MilliQ water at 80 °C at a concentration of 2.5% *w/v*. To the hot dispersion, CaCl_2_ solution was added to get a final salt concentration of 10 or 20 mM. The final concentration of samples was set to 2% *w/v*. Gelation was carried out by letting the samples reach the room temperature. The obtained hydrogels were analyzed by a frequency sweep test carried out at 25 °C at a constant strain of 1% and frequencies ranging from 0.01 Hz to 10 Hz (0.0628 rad/sec to 62.8 rad/sec). A parallel plate geometry with a radial groove and with an 8 mm diameter upper plate was used for the experiments to avoid sample slippage. Each experiment was performed in triplicate.

For stability tests, GG hydrogels were produced as follows: 300 µL of hot aqueous dispersions (80 °C) at 5% *w/v* were poured into a 48 well plate and cooled down to room temperature to induce the sample gelation. The obtained hydrogels were incubated in an orbital shaker incubator at 37 °C with 1 mL of CaCl_2_ 0.1 M for 1 h. After this time, the medium was changed with DPBS pH 7.4 and the samples were further incubated at 37 °C, refreshing the external medium every 72 h. At scheduled time intervals, hydrogel viscoelastic properties were investigated through rheological tests performed in frequency sweep regimes at 25 °C. Strain sweep experiments were preliminarily conducted with an oscillation frequency of 0.1 Hz (0.628 rad/sec) and variable strain percentages from 0.1% to 5% to investigate the linear viscoelastic region. Frequency sweep experiments were conducted at a constant strain of 1% and frequencies ranging from 0.01 Hz to 10 Hz (0.0628 rad/sec to 62.8 rad/sec). A parallel plate geometry with a radial groove and with an 8 mm diameter upper plate was used for the experiments to avoid sample slippage. Each experiment was performed in triplicate.

Morphological analysis was conducted through SEM on freshly prepared samples. For this analysis, hydrogels were washed with MilliQ water to eliminate the salt excess, frozen in liquid nitrogen, cut with a scalpel and freeze-dried.

### 4.6. Hydrogels Swelling and Releasing Ability Studies

The samples prepared as described above were washed in MilliQ water, freeze dried, accurately weighed and incubated in 1 mL of DPBS pH 7.4 at 37 °C in an orbital shaker incubator. At scheduled time points, for the swelling analysis, the excess of medium was eliminated through blotting paper and the swollen sample was weighed again. Swelling percentage (Sw%) was expressed as in the following formula:

Sw% = W_sw_ − W_d_/W_d_ × 100 (1)

where W_sw_ indicates the weight of the hydrogel after swelling and W_d_ indicates the weight of the dry sample. Each experiment was performed in triplicate and the result was expressed as mean value ± standard deviation.

To the samples produced as already described, dextran-FITC was incorporated by mixing with the hot gelling dispersion, an aqueous solution of the fluorescent polysaccharide (1 mg/mL).

The amount of dextran-FITC in each scaffold was 0.4% *w/w* with respect to the GG weight.

Release studies were conducted by submerging hydrogels (each obtained starting from 400 µL of hot gelling dispersion) in 2 mL of DPBS pH 7.4 at 37 °C. After scheduled time points, the release medium was replaced with the same volume of fresh medium and the amount of released dextran-FITC was calculated spectroscopically.

### 4.7. Cytocompatibility Tests and Cell Encapsulation Studies

Preosteoblastic cells MC3T3-E1 were cultured in DMEM medium supplemented with 10% *v/v* of FBS, 1% *v/v* of penicillin–streptomycin solution, 1% *v/v* of glutamine solution and 0.1% *v/v* amphotericin B solution.

Freeze-dried GG samples were sterilized by UV at 254 nm for at least 1 h then dispersed in sterile water at a concentration of 2 mg/mL.

Cells were seeded in 24 well plate (2 *×* 10^4^ per well) and incubated for 24 h in a humidified incubator with 5% CO_2_ atmosphere. A specific volume of GG dispersion was then injected in the supernatant medium to obtain three different concentrations. Cell viability was evaluated after 24 h by means of an MTS assay following the manufacturer’s instructions. Each experiment was performed in triplicate. Viability was expressed as a percentage compared with untreated cells.

For cell encapsulation studies, GG samples were sterilized as just described above and dispersed in sterile water at 2.5% *w/v*. MC3T3-E1 dispersion (100 µL, 1 *×* 10^6^/mL) was mixed with the polysaccharide dispersion (200 µL) into a 1 mL sterile syringe poured into a 48 well culture plate. Cells containing hydrogels were cultured for 72 h by adding 500 µL of culture medium into each culture well. The viability of cells into the constructs were evaluated with a live/dead staining assay following the manufacturer’s instructions.

### 4.8. Statistical Analysis

Swelling data are presented as means ± standard deviation (SD). *t*-test was used for the statistical analysis, *p* values lower than 0.05 were considered as statistically significant and indicated with *.

## Figures and Tables

**Table 1:** The table report molecular weights, polydispersity index and yield % of obtained samples.

Sample	MW (kDa)	PI	Yield %
GG_4_	153(±7.8)	3.03(±0.16)	65
GG_6_	73(±2.1)	2.8(±0.52)	75
GG_8_	59±(4.3)	2.4(±0.23)	70
GG_24_	39±(4.5)	1.7(±0.27)	77

## Data Availability

The data presented in this study are available on request from the corresponding author.
